# Congenital biliary atresia is correlated with disrupted cell junctions and polarity caused by Cdc42 insufficiency in the liver

**DOI:** 10.7150/thno.49116

**Published:** 2021-05-24

**Authors:** Yongjie Zhou, Hongjie Ji, Qing Xu, Xiaoyun Zhang, Xiaoyue Cao, Yuwei Chen, Mingyang Shao, Zhenru Wu, Jie Zhang, Changli Lu, Jiayin Yang, Yujun Shi, Hong Bu

**Affiliations:** 1 Laboratory of Pathology, Key Laboratory of Transplant Engineering and Immunology, NHC, West China Hospital, Sichuan University, Chengdu 610041, China.; 2 Department of Pathology, West China Hospital, Sichuan University, Chengdu 610041, China.; 3 School of Bioscience and Technology, Weifang Medical University, Weifang 261042, China.; 4 Department of Liver Surgery, West China Hospital, Sichuan University, Chengdu 610041, China.; 5 Core Facility of West China Hospital, Sichuan University, Chengdu 610041, China.

**Keywords:** Cdc42, biliary atresia, epithelial junction, cell polarity, bile acids

## Abstract

**Rationale:** Congenital biliary atresia (BA) is a destructive obliterative cholangiopathy of neonates that affects both intrahepatic and extrahepatic bile ducts. However, the cause of BA is largely unknown.

**Methods:** We explored the cell junctions and polarity complexes in early biopsy BA livers by immunofluorescence staining and western blot. Cdc42, as a key cell junction and polarity regulator, was found dramatically decreased in BA livers. Therefore, in order to investigate the role of Cdc42 in BA development, we constructed liver-specific and tamoxifen induced cholangiocyte-specific Cdc42 deleted transgenic mice. We further evaluated the role of bile acid in aggravating biliary damage in Cdc42 insufficient mouse liver.

**Results:** We found a dramatic defect in the assembly of cell junctions and polarity complexes in both cholangiocytes and hepatocytes in BA livers. This defect was characterized by the disordered location of cell junction proteins, including ZO1, β-catenin, E-cadherin and claudin-3. Cdc42 and its active form, Cdc42-GTP, which serves as a small Rho GTPase to orchestrate the assembly of polarity complexes with Par6/Par3/αPKC, were substantially reduced in BA livers. Selective Cdc42 deficiency in fetal mouse cholangiocytes resulted in histological changes similar to those found in human BA livers, including obstruction in both the intra- and extrahepatic bile ducts, epithelial atrophy, and the disruption of cell junction and polarity complexes. A reduction in bile acids notably improved the histology and serological indices in Cdc42-mutant mice.

**Conclusion:** Our results illustrate that BA is closely correlated with the impaired assembly of cell junction and polarity complexes in liver cells, which is likely caused by Cdc42 insufficiency and aggravated by bile acid corrosion.

## Introduction

Congenital biliary atresia (BA) is a destructive inflammatory obliterative cholangiopathy of neonates that influences varying lengths of both intrahepatic and extrahepatic bile ducts, and this disorder affects 1 in 8000-18000 live births 1. Although the cause of BA is largely unknown, some factors, such as genetic, infective, toxic and inflammatory factors, are thought to contribute to its development 2. Among these factors, inflammation and viral infection have long been believed to be the main cause of BA 3. Evidence supporting this hypothesis originates from the serological parameters of BA and the similarity in the pathological features between patients with BA and patients infected with viruses. This hypothesis is further supported by findings that newborn BALB/c mice intraperitoneally inoculated with rhesus rotavirus (RRV) strains or SA11-FM gradually develop BA-like obstruction of the extrahepatic bile ducts 4. The deletion of INFγ, IL-12 or IL-33 blocks viral-induced BA development in these animals 5-7. However, the great advances in antiviral, including rotavirus vaccination tested in the United States and Korea 8, 9, and anti-immune therapies achieved in recent decades have not effectively relieved or prevented the occurrence of BA, which indicates that viral infection and immune disorders might be secondary events occurring in livers that are susceptible to damage or that harbor basic lesions caused by genetic dysregulation.

Increasing evidence indicates that BA patients have a genetic susceptibility that makes them more prone to toxic insults and enhanced biliary injury. Several genome-wide association studies (GWAS) have revealed that many susceptible candidate genes, including *ADD3*, *ARF6* and *GPC1*, are the most frequently mutated genes 10-14. ADD3 belongs to a family of membrane skeletal proteins involved in the assembly of spectrin-actin networks at sites of cell-cell contact in epithelial tissues; ARF is a GTP-binding protein that regulates vesicle transport and cytoskeletal remodeling; and GPC1 is a proteoglycan that exhibits an apical distribution in the liver 12. Studies have clearly shown that all of these proteins are associated with cell-cell junctions or cell polarity, and knockdown of any of these proteins leads to defects in the biliary system in zebrafish, which suggests that aberrant cell junctions or cell polarity might be associated with BA 13, 14. However, the frequencies of mutations in the* ADD3* and *ARF6* genes are only very slightly elevated in patients (the frequencies in patients are 53.9% and 28.6%, whereas those in the general population are 41.7% and 13.1%, respectively). In addition, *GPC1* is altered in a very small proportion of BA patients (2/35) 12. Although previous studies have revealed that dozens of genes likely play roles in the development of BA, the driver gene(s) remains obscure. Nevertheless, the mutation of genes that encode proteins involved in cell junctions or cell polarity is a suspect in this pathological process.

Epithelial cells form single- or multilayered sheets that cover all body surfaces and cavities and constitute the parenchyma of glandular organs 15. The formation of epithelial layers with apicobasal polarity is a fundamental developmental process. The polarity is reflected by the division of the plasma membrane into the following two surfaces: an apical surface facing the lumen and a basolateral surface that contacts adjacent cells and the underlying extracellular matrix (ECM). This division is accomplished and maintained by specialized junctional complexes comprising tight junctions (TJs) and adherens junctions (AJs) 15. TJs establish a selective paracellular permeability barrier and play a substantially more important role in separating the apical portion from the basolateral portion of the cellular membrane, whereas AJs are responsible for cell-cell adhesion and play a key role in tissue sorting during development through the expression of specific cadherin isoforms. Both TJs and AJs are focal sites for the anchoring and organization of the cytoskeleton, the disruption of which results in severe abnormalities. For example, the deletion of ZO1 or ZO2, which are two of the cytoplasmic domains of TJs, resulted in embryonic lethality 16, 17.

Both junctional and polarity-related proteins play fundamental roles in the maintenance of the architecture and function of the biliary tree. Bile is secreted by the liver into small ducts that join to form the common hepatic duct. The biliary epithelium, which is connected by junctional proteins, forms a barrier that protects hepatocytes and the epithelium itself from bile corrosion. Alterations in AJs and TJs are implicated in chronic cholestatic liver diseases, such as primary biliary cirrhosis and primary sclerosing cholangitis 18, 19; biliatresone, a newly identified toxin, can disrupt TJs in cultured cholangiocyte spheres and lead to extra-hepatic bile duct injury and BA-like histopathologic changes *in vivo* 20-22; however, it remains unknown whether defects in cell junctions and polarity are commonly present in human BA liver.

Cdc42 is a member of the small Rho GTPase family that regulates cell polarity across organisms from yeast to humans 23, 24. Cdc42 is a pivotal regulator of polarized morphogenesis in epithelial cells, through coordination of apical membrane morphogenesis, lumen formation and junction maturation. Increasing evidence reveals that organ specific loss of Cdc42 expression leads to severe developmental defects in pancreas, skin, kidney and lung 25-28. In addition, Cdc42 also plays important roles in tumor cell proliferation and migration 29, 30. We found severely disrupted cell junction and polarity complexes in BA livers, which might be correlated with defects in the Cdc42-Par6/Par3/αPKC complex, a critical complex in establishing cell polarity 31. The role of Cdc42 in BA development remains unknown. In the present study, by inducibly ablating the Cdc42 gene in cholangiocytes, we reproduced BA pathological changes in the livers of newborn mice. This model was found to be more suitable than the established mouse model induced by RRV in reflecting BA pathology. Furthermore, our work provides insight into the role of bile acids in disrupting the epithelial barrier in livers harboring genetic dysregulation.

## Materials and methods

### Human BA samples

BA liver samples were collected from fourteen infant patients (6 males and 8 females, at an age of 1.62 ± 0.19 months) when they underwent a diagnostic laparoscopic exploration. Their liver specimens were termed as early-stage BA. Five resected BA livers at liver transplantation were considered as end-stage BA; these patients were 2.5-4.8 years old. Liver tissues obtained from three children aged 1 to 2 months who died from non-liver diseases were used as a normal control. Fresh tissue was used for protein and nucleic acid extraction, while paraffin-embedded tissue was subjected to histological examination. Written informed consent was obtained prior to sample collection. The procedures used for human sample collection and use were approved by the ethics committee of West China Hospital, Sichuan University.

### Animals

*Cdc42^loxP/loxP^* (*Cdc42^fl/fl^*) C57bl6 mice were purchased from The Jackson Laboratory 32. Keratin 19-CreERT (K19-CreERT) transgenic mice (C57bl6) were purchased from the Model Animal Research Center of Nanjing University (Nanjing, China) 33. Liver-selective Cdc42-ablated mice (*Alb-Cre:Cdc42^-/-^*) were generated by intercrossing an Alb-cre C57bl6 mouse with a *Cdc42^loxP/loxP^* mouse. *K19-CreERT* mice were crossed with *Cdc42^loxP/loxP^* mice and recombination was induced by tamoxifen (*K19-CreERT:Cdc42^-/-^*). Mice without Cre recombinase or tamoxifen administration were considered as wild type controls.

To establish an RRV-induced mouse model of BA, newborn BALB/c mice were intraperitoneally inoculated with 25 μL of minimum essential medium containing 10^6^ PFU of RRV 34. All mice were observed for signs of cholestasis for 14 days, at which point they were euthanized. Liver samples were collected for histological analysis.

All mice used in this study were fed a normal chow diet and housed with corncob bedding under SPF conditions. The animal handling and care procedures were conducted in accordance with national and international laws and policies and were approved by the Animal Care and Use Committee of Sichuan University.

### Immunoprecipitation, GST-PBD pull-down assay and western blotting

Co-IP was performed using the Catch and Release v2.0 Reversible Immunoprecipitation System (#17-500, Millipore, USA) according to the manufacturer's instructions. To detect the components of the polarity complex, Par6 antibody-coded beads (#ab180159, Abcam, USA) were used to pull down Par6 protein and proteins bound to Par6 from liver homogenates. Cdc42 (#ab41429, Abcam), Par3 (#NBP1-88861, Novus, USA) and αPKC (#ab32376, Abcam) were separately examined. The GST-PBD pull-down assay was carried out using the Cdc42 Activation Assay Biochem Kit™ according to the manufacturer's instructions (#BK034-S, Cytoskeleton, Colorado, USA). Briefly, tissue samples were homogenized on ice in cell lysis buffer (50 mM Tris-HCl [pH 7.4], 200 mM NaCl, 5 mM MgCl_2_, 1% [vol/vol] Nonidet P-40, and 10% [vol/vol] glycerol) with 1 mM phenylmethylsulfonyl fluoride and 2 mM Na_3_VO_4_. Equal amounts of protein lysates were incubated with 8 μg of GST-PBD (amino acids 69 to 150 of human PAK1) for 4 hours at 4 °C and washed, as previously reported 35. The bead pellet was suspended in 20 μL of Laemmli sample buffer. Proteins were separated *via* 12% sodium dodecyl sulfate-polyacrylamide gel electrophoresis and analyzed by western blotting. Liver tissue samples were lysed in RIPA buffer and centrifuged at 13,000 rpm for 15 min at 4 °C. Western blotting was performed using standard protocols. The antibodies and regents used in this study are listed in Supplementary Table 1 and Supplementary Table 2. The proteins were normalized by the gray value measured by Image J software.

### Whole-exome sequencing and pathway analysis

Whole-exome sequencing of nine human BA livers and the data analyses were performed by Guangzhou RiboBio Co., Ltd. (China). Mutations were identified by comparisons with the 1000Genome, dbSNP138 and ESP6500 databases, and PhyloP and SIFT were used to predict the functional effects of missense mutations. The mutual correlation of specific proteins (protein-protein-interaction, PPI) was analyzed using the online platform String (https://string-db.org/). The sequencing data can be provided by the corresponding author upon reasonable request.

### Statistical analysis

The statistical analyses were performed using SPSS 19 or Prism GraphPad 7. The results are expressed as the means ± s.e.m. The significance of the differences between groups was tested using an unpaired two-tailed Student's *t* test with Welch's correction. A *P*-value < 0.05 was considered significant.

For more details, see the Supplementary Material.

## Results

### Disruption of epithelial junctions and cell polarity in BA livers

**Figure 1:**
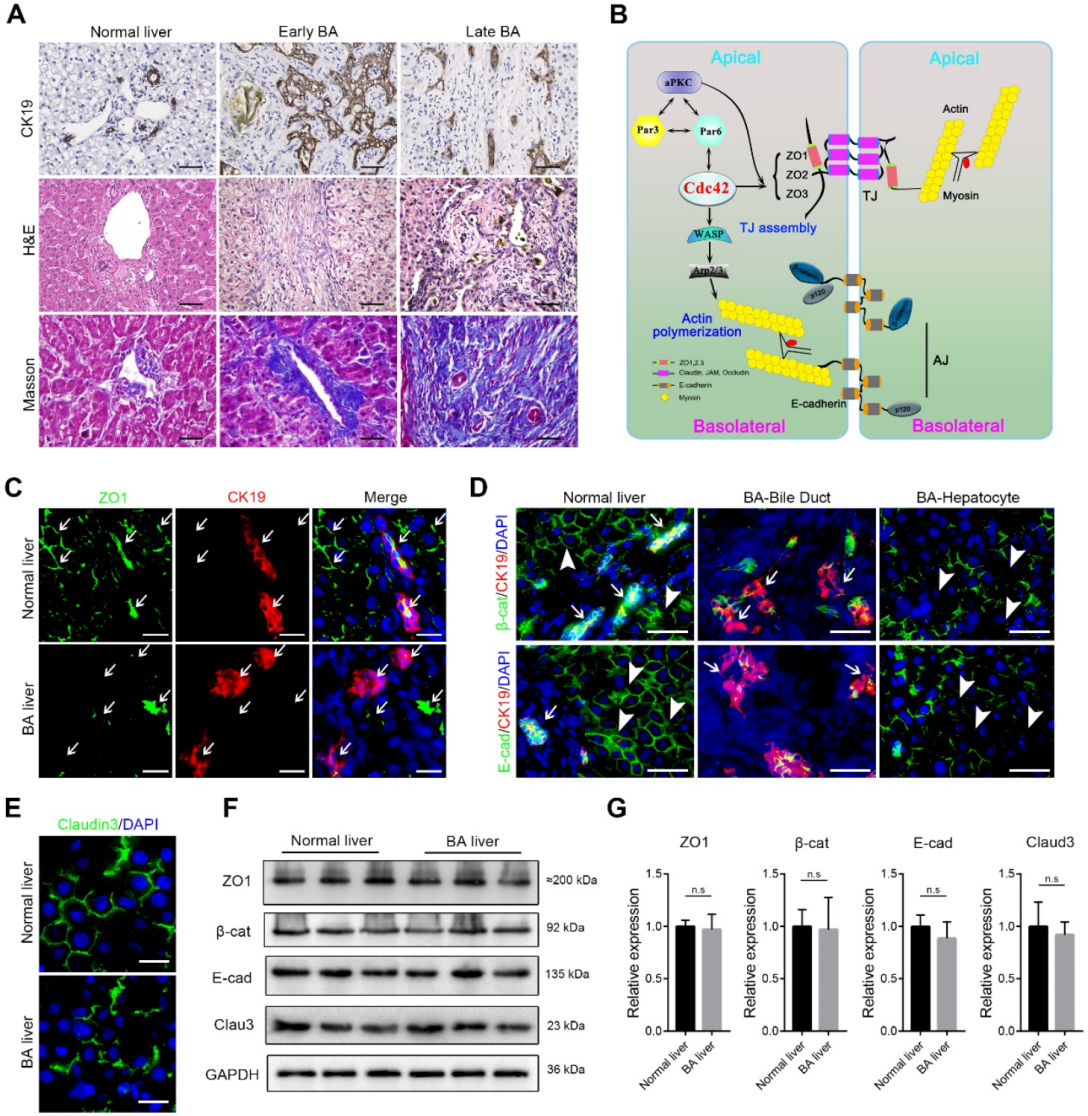
** Disruption of junction and polarity proteins in the BA liver. (A)** Ductal reaction, inflammatory cell infiltration and collagen deposition in early and late BA measured by CK19 immunohistochemistry staining, H&E staining and Masson trichrome staining, respectively. Scale bar, 100 µm.** (B)** Simplified diagram of the composition of cell junction and polarity complexes. **(C)** Representative images of dual-immunofluorescence staining of the tight junction protein ZO1 and the cholangiocyte marker CK19 in normal and BA liver sections; aberrant ZO1 staining was detected in all BA patient livers (14/14). Arrows indicate the small bile ducts. Scale bar, 50 µm. **(D)** Immunofluorescence detection of the adherens junction proteins E-cadherin and β-catenin in combination with CK19 in normal and BA liver sections. Note that E-cadherin and β-catenin are aberrantly distributed in both cholangiocytes (arrows) and hepatocytes (arrowheads) in BA livers (14/14). Scale bar, 100 µm. **(E)** Immunofluorescence staining of the tight junction protein claudin-3 in normal and BA liver sections; claudin-3 was aberrantly expressed in all the BA patient livers examined (14/14). Scale bar, 50 µm. **(F)** Immunoblotting was performed using total protein lysates from three normal livers and three BA livers. **(G)** The expression of each protein was further semiquantified using the ImageJ software; n.s, not significant.

Typical histological changes including malformed bile ducts, shrunk lumen, atrophy or vanished epithelium, as well as biliary atresia and ductal hyperplasia were prominently observed in the early BA livers. Of note, in type I BA, which is considered to mainly involve the extrahepatic bile duct, the hierarchical intrahepatic bile ducts were substantially impaired. Destruction of the liver resulted in the accumulation of extracellular matrix and the recruitment of a considerable amount of inflammatory cells to the portal triads. In the end-stage BA livers, the biliary epithelium was nearly absent and the lumen was severely narrowed. The liver parenchyma was moderately to severely destroyed and replaced by fibrous tissues (Figure 1A).

The correct assembly of junction and polarity proteins is necessary for the formation and maintenance of the epithelial barrier (Figure 1B) 15, 24. To address whether epithelial junctions and cell polarity are associated with BA, we examined the expression of key junction proteins in liver tissue biopsies obtained from early-stage BA patients. ZO1, a key TJ protein that participates in TJ assembly and forms a barrier to protect cells from stress, was constitutively and regularly distributed in surrounding hepatocytes and on the lumen surface of the bile duct in the normal liver. In contrast, in all (14/14) examined BA patient livers, ZO1 was either absent in most parenchymal cells or showed fragmented and plaque-like accumulation (Figure 1C). Similarly, β-catenin, E-cadherin and claudin-3, all of which are critical AJ and TJ proteins, showed substantial disruption and irregular accumulation around hepatocytes and cholangiocytes (Figure 1D-E). Because both hepatocytes and cholangiocytes were found to have aberrant expression of AJ and TJ proteins, we performed immunoblotting to examine the expression of these proteins in the whole liver homogenates. Surprisingly, in contrast to the immunofluorescence staining results, the western blotting assay did not show these junction proteins to be markedly decreased in the BA liver (Figure 1F), which indicated that these cell junction- and polarity-related proteins were more likely misassembled rather than having a defect in protein production.

### Cdc42 is robustly dysregulated in BA livers

**Figure 2:**
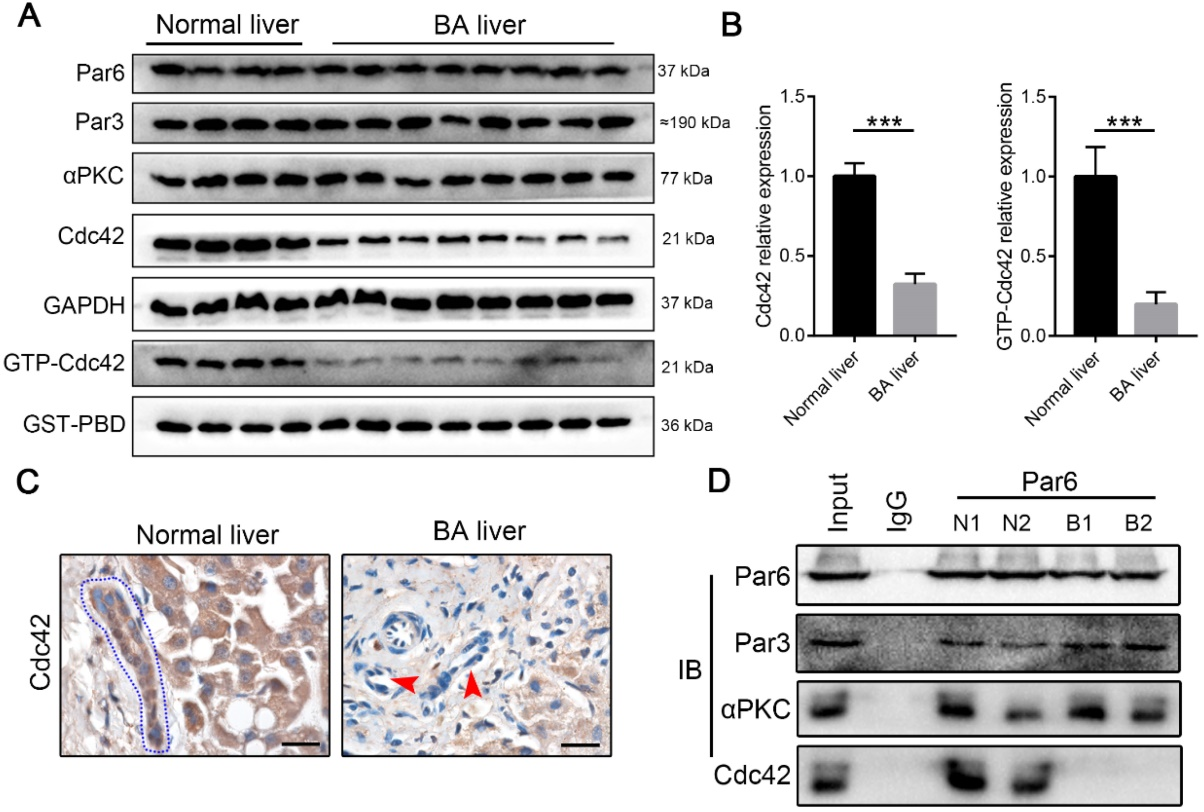
** Cdc42 is substantially reduced in the BA liver. (A)** Immunoblotting assays were performed to analyze the expression of Par3/Par6/αPKC/Cdc42 complex proteins, and a GST-PBD pull-down assay was performed to determine the expression of active GTP-Cdc42, in normal livers (n=4) and BA livers (n=8). **(B)** The levels of Cdc42 and GTP-Cdc42 in normal and BA livers are expressed as ratios relative to GAPDH and GST-PBD expression, respectively. ***, *P* < 0.001. The data are presented as the means ± s.e.m.; the *P* value was determined by the two-tailed Student's t test. **(C)** Cdc42 staining in normal (n=4) and BA liver sections (n=14). Cholangiocytes in normal liver are marked by a blue dotted circle. Arrowheads indicate the deficient Cdc42 expression in the BA liver, particularly in cholangiocytes. Scale bar, 20 µm. **(D)** A coimmunoprecipitation assay showed that Cdc42 does not bind to Par6 in the BA liver.

The components of the Par3/Par6/αPKC/Cdc42 polarity complex, particularly Cdc42, are indispensable for the establishment of epithelial cell polarity 22-24. Western blotting demonstrated that BA livers exhibited a very slight change in Par6, Par3 and αPKC expression, but they had a notable decrease in Cdc42 expression compared with normal neonatal livers (Figure 2A-B). Parallel to the total Cdc42, in BA livers the active form of Cdc42, Cdc42-GTP, showed a greater than two-fold decrease (Figure 2A-B). Immunohistochemistry staining again illustrated Cdc42 deficiency in BA livers, particularly in cholangiocytes (Figure 2C).

Distinct from other small Rho GTPase family members, Cdc42 has the specific ability to orchestrate the assembly of polarity complexes with Par6/Par3/αPKC and thus plays a central role in the regulation of cell junctions and polarity 31, 36. An immunoprecipitation assay showed that Par6, Par3, αPKC and Cdc42 formed a complex in the normal liver, whereas Par6, Par3 and αPKC were assembled together without Cdc42 in the BA liver (Figure 2D). It has been well documented that Cdc42 plays a pivotal role in tubulogenesis and organogenesis by regulating the epithelial polarity in various organs 25-28; therefore, the defect of Cdc42 in the polarity complex is highly likely to be associated with BA development.

### Mouse livers with selective Cdc42 ablation in cholangiocytes develop human BA-like changes

**Figure 3:**
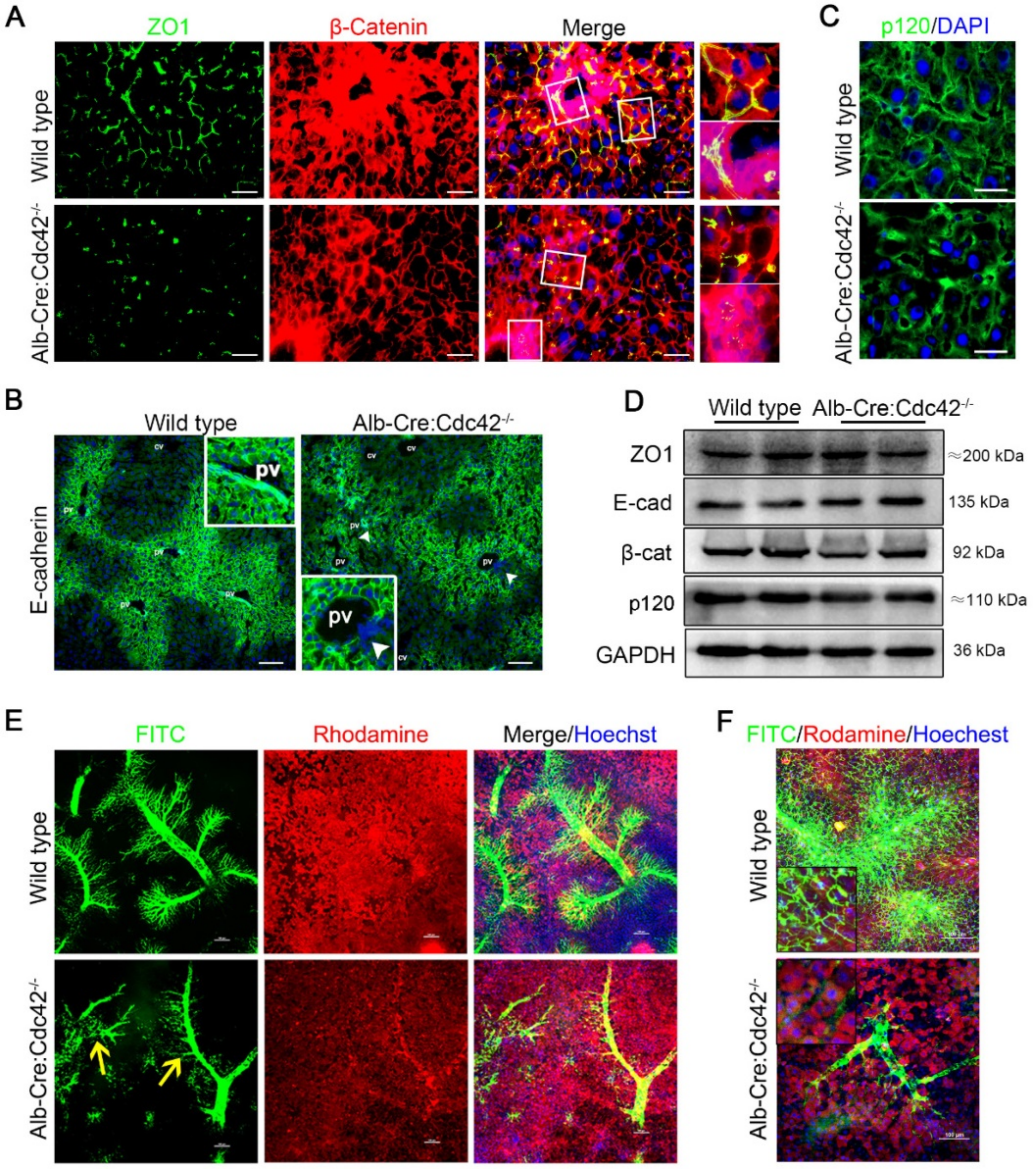
** Liver-specific Cdc42 deletion leads to severe cell junction and polarity disruptions and intrahepatic biliary tract obstruction. (A)** Liver sections from 2-month-old wild type (n=5) and *Alb-Cre:Cdc42^-/-^* (n=10) mice were stained with antibodies against ZO1 (green) and β-catenin (red). The insets on the right depict a higher-magnification image of the boxed area. Scale bar, 50 µm. **(B)** Liver sections (n=10) were stained with E-cadherin antibody. The insets depict a higher-magnification image, and white arrowheads indicate disordered expression of E-cadherin. Scale bar, 100 µm. PV, portal vein; CV, central vein. **(C)** Immunofluorescence staining of p120 in liver sections (n=10). Scale bar, 25 µm. **(D)** Immunoblotting showed no obvious differences in ZO1, E-cadherin, β-catenin or p120 expression between the wild type and *Alb-Cre:Cdc42^-/-^* livers. **(E)** The intrahepatic bile tracts were clearly visualized by retrograding FITC solution (green), and the surrounding hepatocytes and nuclei were labeled with rhodamine (red) and Hoechst (blue), respectively. The yellow arrows indicate disrupted or misarranged bile tracts in the *Alb-Cre:Cdc42^-/-^* liver. Scale bar, 100 µm. **(F)** With the increase of infused FITC solution, the end-terminal ductules (canaliculi) were easily visualized in the normal liver; however, there were prominent filling defects in the small ductules in the BA liver. Scale bar, 100 µm.

Consistent with a previous study in *Alb-Cre:Cdc42^-/-^* mice 37, we found that Cdc42 deficient liver exhibited histological changes including biliary tract injury, fibrosis and inflammatory infiltration (Figure S1-2). The cell junctions were severely disrupted, which was similar to the situation in BA patients. Immunofluorescence staining showed that ZO1 protein was prominently discontinuous (Figure 3A). E-cadherin, one of the most important AJ proteins that provides binding sites to or acts as a platform for other AJ proteins, such as p120 and β-catenin, showed a clear spatial distribution and was specifically highly expressed in the periportal area in wild type mice. However, the spatial distribution of E-cadherin was obscure in Cdc42-deficient livers and was even absent in some periportal cells (Figure 3B). β-catenin and p120 were also disarranged in Cdc42-deficient livers (Figure 3A, C). Of note, although the immunofluorescence demonstrated that the junction and polarity proteins were aberrantly distributed, their relative expression levels were not significantly altered (Figure 3D). These findings strongly highlighted the fundamental role of Cdc42 in orchestrating the correct assembly and distribution of junction and polarity complexes.

Using a confocal microscopy technique and retrograde perfusion of fluorescent dyes via the common bile duct, we visualized the hierarchical structure of the 2-month-old mouse biliary tree at a fine-scale, as previously described 38. In brief, when the fluorescent dyes Rhodamine B (red) and Hoechst (blue) are intraperitoneally injected, they are absorbed by live hepatocytes and cholangiocytes and can clearly distinguish the liver cells and nuclei, respectively. Rhodamine is then excreted into the bile and mixed with retrogradely infused FITC (green), making the bile duct appear to be filled with yellow fluorescent liquid under a confocal microscope. As shown in Figure 3E, in the wild type liver, the hierarchical structure of the intrahepatic biliary tracts was well visualized. In particular, the connections between terminal bile ducts (bile canaliculi) outlined each hepatocyte (Figure 3F). In contrast, in the Cdc42-mutant liver, the smaller ducts and the bile canaliculi were very poorly visualized, suggesting their obstruction and adding evidence that a Cdc42 “defect” result in abnormal bile duct development and bile duct damage is secondary to abnormal bile ducts (Figure 3E-F).

**Figure 4:**
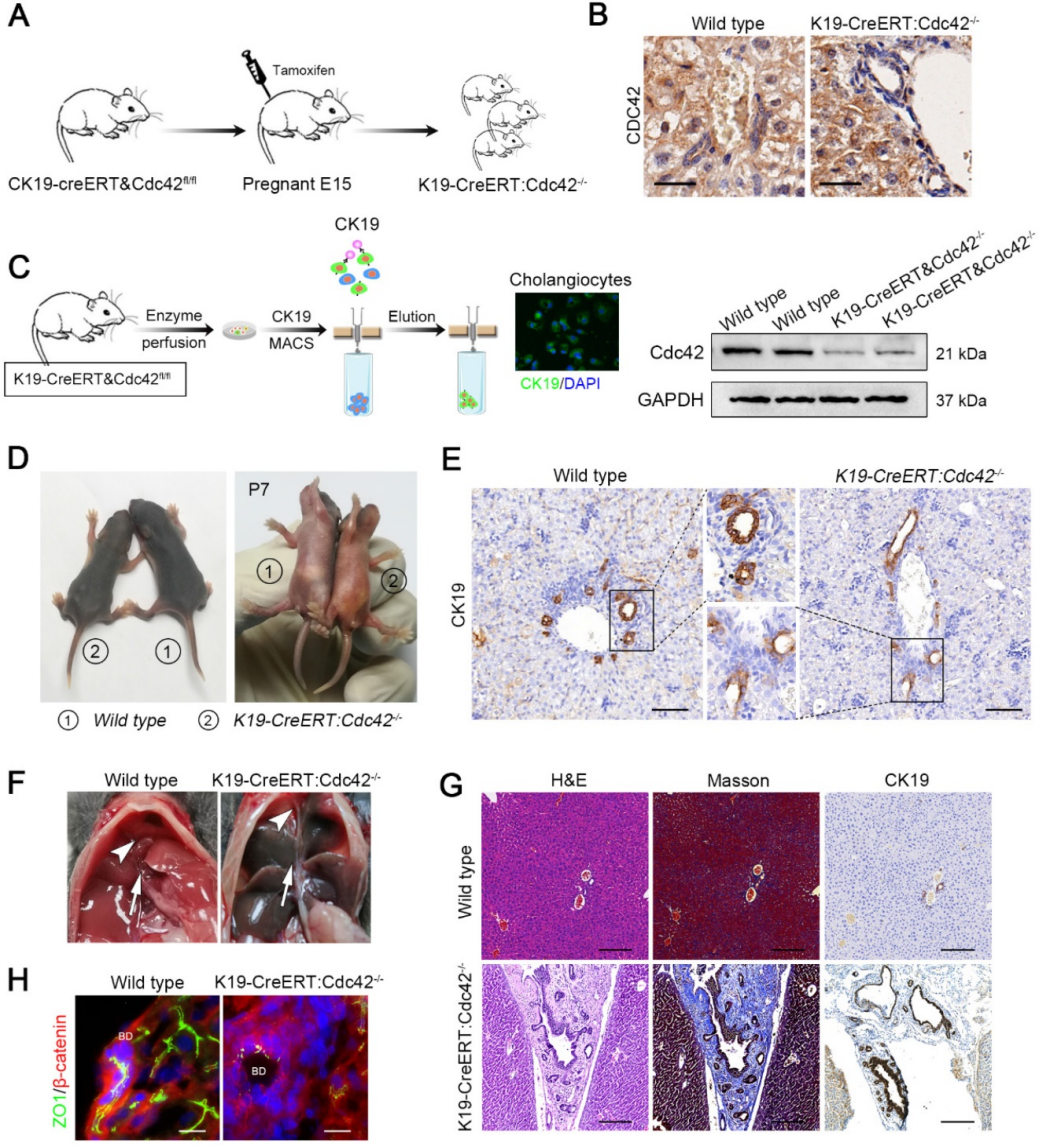
** Inducible cholangiocyte-specific Cdc42 ablation leads to liver injury and biliary tract obstruction in postnatal mice. (A)** Simplified diagram depicting the strategy for the development of tamoxifen-induced cholangiocyte-specific Cdc42-ablated fetal mice. **(B)** Cdc42 staining of liver sections (n=6) from 4-week-old mice confirmed the specific Cdc42 ablation in biliary epithelial cells (highlighted by red dotted circles). Note the disarrangement of the biliary epithelium. Scale bar = 20 µm. **(C)** Cholangiocyte isolation strategy and knockout efficiency confirmed by immunoblotting assay. **(D)** Macroscopic views of wild-type and *K19-CreERT:Cdc42^-/-^* mice at 7 days after birth. **(E)** CK19 immunohistochemistry staining of P7 mouse lives sections (n=5). **(F)** Anatomical views of gallbladder (white arrowhead) and extrahepatic bile ducts (white arrow) of wild type and *K19-CreERT: Cdc42^-/-^* mice. **(G)** H&E, Masson trichrome and CK19 immunohistochemistry staining of wild type and *K19-CreERT:Cdc42*^-/-^ liver at four weeks of age (n=5). Scale bar, 200 µm. **(G)** ZO1 and β-catenin staining showed cell junction disruption caused by Cdc42 ablation (n=5). Scale bars, 25 µm.

Notably, the phenotype in this model is in contrast to the clinical situation in which extrahepatic bile ducts are more often and more severely involved than intrahepatic ducts. This might be because hepatoblasts begin to produce albumin from E8.5 39 and then trigger the *Alb-cre/loxp* system-based gene ablation; however, the differentiation of hepatoblasts into hepatocytes and cholangiocytes occurs around E15 40, and as a result, their progeny cells, including hepatocytes and intrahepatic bile epithelial cells, are all deprived of target gene expression. In contrast, the extrahepatic bile duct shares the same germinal layer origin as the pancreas, which is different from the germinal layer origin of the intrahepatic biliary system; therefore, the target gene in extrahepatic bile ducts and pancreatic ducts could remain intact. We next investigated whether selective Cdc42 ablation in cholangiocytes leads to biliary epithelial injury, particularly in extrahepatic bile ducts. We next generated *K19-CreERT:Cdc42^-/-^* mice and their Cdc42 was inducibly ablated by single dose of intraperitoneal tamoxifen injection in the pregnant mice on E15. A total of fifteen pregnant mouse mice were intraperitoneally injected with a single dose of tamoxifen; all genotypic mice were born normally in accordance with Mendelian law without obvious difference in litter size. As a result, the expression of Cdc42 in the total biliary system was successfully deleted in the neonatal mice (Figure 4A-C).

As expected, over three-fourths (19/25) neonatal mice at P7 displayed growth retardation and jaundice (Figure 4D) and the mice without apparent abnormal phenotype were excluded for further evaluation. The bile ducts of *K19-CreERT:Cdc42^-/-^* mice at P7 were abnormally organized as indicated by CK19 staining (Figure 4E). Correspondingly, serological liver indices as well as the reported BA-related inflammatory factors, such as IFNγ, IL-2, TNFα and IL-17A 3 were highly elevated in the *K19-CreERT:Cdc42^-/-^* mice (Figure S3). The mice were sacrificed at the age of 6 weeks. Under a gross view, the diseased liver was dark-yellow in color. Because the gallbladder and extrahepatic bile ducts were extremely atrophied (Figure 4F), we failed to conduct retrograde cholangiography with dye injection. Microscopically, similar to the changes in the human BA liver, the hilar bile duct lumen was obviously narrow and irregular, and the epithelium was atrophic or absent. Most prominently, the atrophic bile ducts, along with the reactively proliferated ducts, were entrapped in very thick fibrous scars (Figure 4G). And the EHBD was apparently obstructed in *K19-CreERT:Cdc42^-/-^* mice (Figure S4). As expected, ZO1 was aberrantly distributed in the cholangiocytes (Figure 4H). Finally, 40% (6/15) of the *K19-CreERT:Cdc42^-/-^* mice died before 2 months of age.

### Bile acids aggravate biliary injury when Cdc42 is insufficient

**Figure 5:**
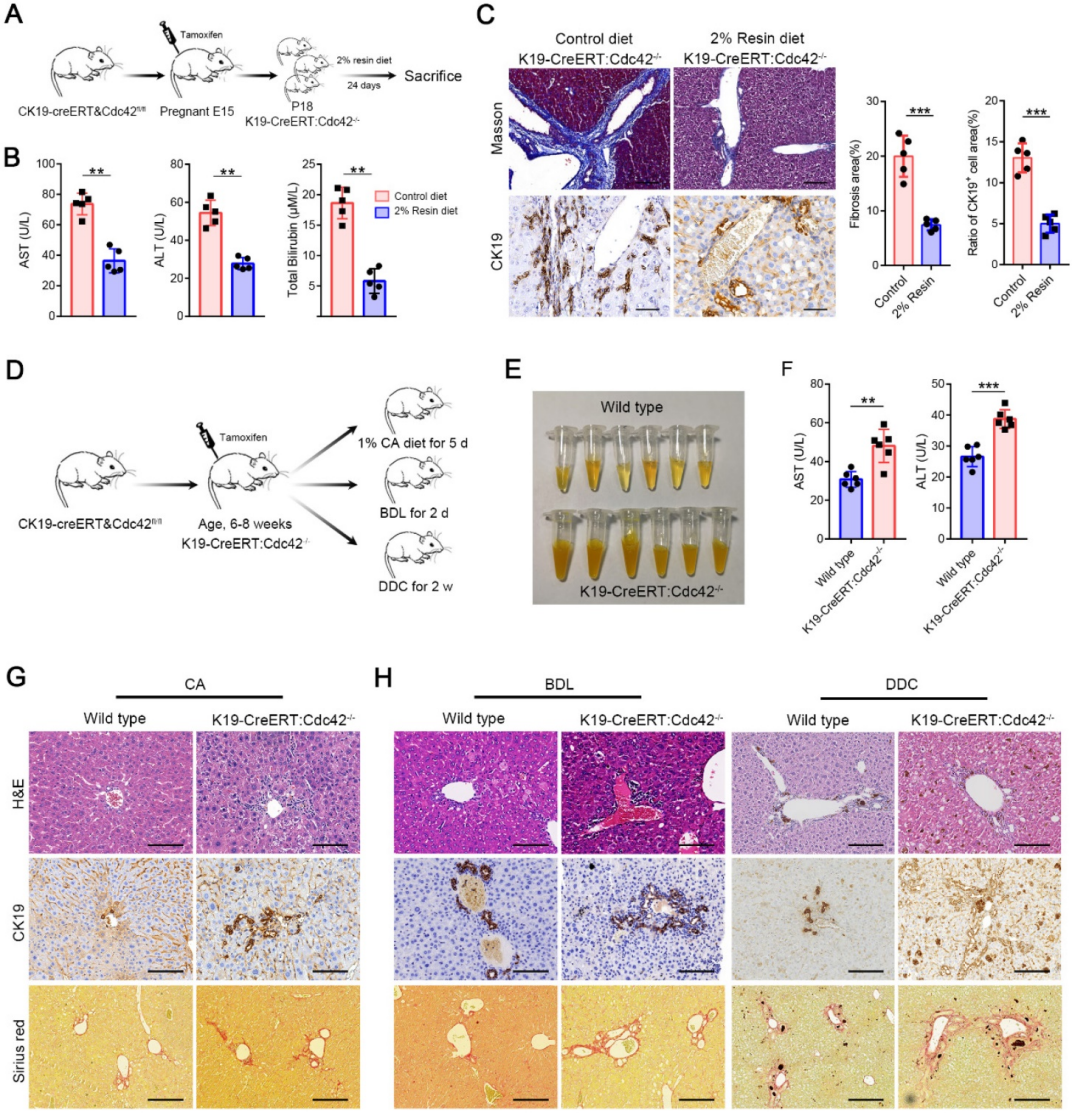
** Bile acids aggravate biliary injury in *K19-CreERT:Cdc42^-/-^* mouse liver**. **(A)** Simplified diagram depicting the strategy for the evaluating the effects of reducing bile acids on *K19-CreERT:Cdc42^-/-^* mouse liver. **(B)** Hepatic enzymes of 6-week-old mice were measured after feeding with a 2% resin diet (n=5), **, *P* < 0.01. The data are presented as the means ± s.e.m.; the *P* value was determined by the two-tailed Student's t test. **(C)** The fibrosis and ductal reaction measured by Masson trichrome and CK19 immunohistochemistry staining (n=5), respectively. Scale bar, 200 µm (Masson) and 100 µm (CK19). ***, *P* < 0.001. The data are presented as the means ± s.e.m.; the *P* value was determined by the two-tailed Student's t test. (D) Simplified diagram depicting the strategy for the evaluating the effects of excessive bile acids or cholangiocyte targeting model on *K19-CreERT:Cdc42^-/-^* mouse liver. (E) The serum color of the wild type and *K19-CreERT:Cdc42^-/-^* mouse after cholic acid feeding (n=6). (F) Hepatic enzymes of 6-week-old mice were measured after feeding with a 1% cholic acid diet (n=6), **, *P* < 0.01. The data are presented as the means ± s.e.m.; the *P* value was determined by the two-tailed Student's t test. (G-H) Histologic alteration, ductular reaction and liver fibrosis were measured by H&E staining, CK19 immunohistochemistry staining and sirius red staining in cholic acid diet feeding model (G), BDL and DDC models (H). Scale bar, 100 µm, 100 µm and 200 µm, respectively.

The symptoms and signs of BA occur very shortly after birth, but the histological features of BA have never been measured in aborted fetuses. Moreover, the pancreatic ducts are seldom reported as being involved in BA patients, which share the same cell origin and cell marker (for example, CK19) with extrahepatic cholangiocytes. Interestingly, in *K19-CreERT:Cdc42^-/-^* mice, the pancreatic ducts were simultaneously deprived of Cdc42 expression and displayed secondary aberrant distribution of ZO1, E-cadherin and β-catenin (Figure S5). Thus, we investigated whether the bile acids cause biliary damage if epithelial cells have a preexisting abnormal structure, because bile acid is greatly increased in newborns by feeding. We fed the weaned *K19-CreERT:Cdc42^-/-^* mice (at an age of ~18 days) with a chow diet supplemented with 2% cholestyramine resin for 24 days, which significantly decreases bile excretion by preventing the enterohepatic circulation of bile acids 41. The effective reduction of bile acids notably improved the serological indices, and dramatically reduced liver fibrosis and cholangiocyte proliferation in the *K19-CreERT:Cdc42^-/-^* mice (Figure 5A-C). Conversely, in order to evaluate whether excessive bile acid can aggravate liver injury when Cdc42 is insufficient, adult *K19-CreERT:Cdc42^-/-^* mice were treated with tamoxifen injection and then subjected to 1% cholic acid supplemented diet for 5 days (Figure 5D) 42. Even though inducible Cdc42 deletion in mature mouse did not rouse obvious biliary tract injury (Figure S6), more severe liver injury was observed in* K19-CreERT:Cdc42^-/-^*mice after intaking excessive bile acid (Figure 5E-G). Similar phenomena were also observed in another two mouse models treated with a surgery of bile duct ligation (BDL) or a diet with 0.1% 3,5-diethoxycarbonyl-1,4-dihydrocollidine (DDC) (Figure 5D, H), both of which are characterized by biliary injury 43.

### Bile acids do not significantly impair Cdc42 expression, cell junctions or polarity

**Figure 6:**
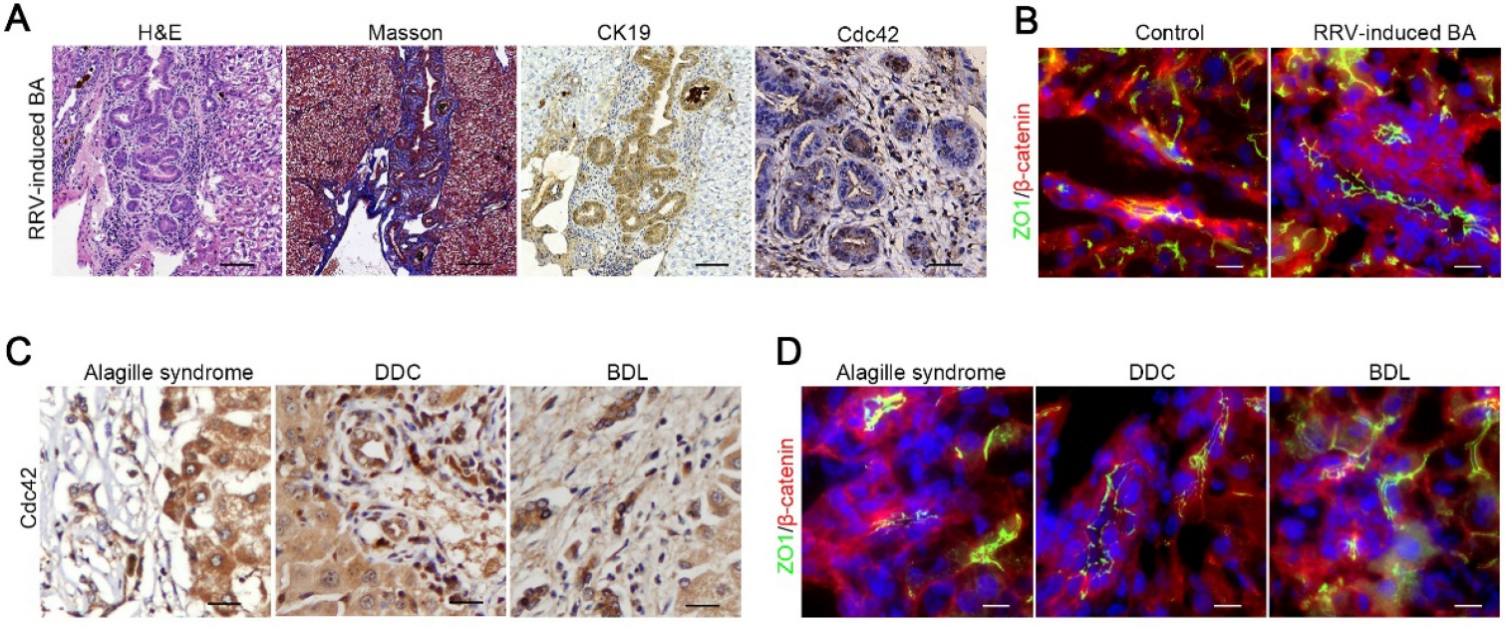
** Bile acids have little effect on Cdc42 expression**. **(A)** Histological alteration and Cdc42 expression in RRV-induced BA model (2-week-old mice) (n=6). Scale bar, 500 µm (H&E), 200 µm (Masson and CK19) and 50 µm (Cdc42). **(B)** ZO1 and β-catenin staining of control (n=3) and RRV-induced BA sample (n=6). Scale bars, 25 µm. **(C)** Cdc42 staining of Alagille syndrome samples (5/5) and DDC or BDL mouse liver samples (n=6). Scale bar, 50 µm. **(D)** Immunofluorescent staining of ZO1 and β-catenin in an Alagille syndrome sample and DDC or BDL mouse liver samples. Scale bar, 25 µm.

Our data indicated that bile acids potently damage Cdc42-deficient cholangiocytes; thus, we next investigated whether bile retention, in turn, impairs Cdc42 expression in biliary cells. We first established an RRV-induced BA mouse model that displayed similar cholangiocyte injury and bile retention. In contrast to human BA liver and our *K19-CreERT:Cdc42^-/-^* liver, Cdc42 was strongly expressed in RRV-induced BA liver (Figure 6A). Correspondingly, cell junction and polarity proteins, such as ZO1 and β-catenin, were normally expressed on the surface of cholangiocytes (Figure 6B).

Next, we performed immunohistochemistry staining on tissue samples from an obstructive jaundice human liver secondary to Alagille syndrome, and mouse livers treated with DDC diet or BDL surgery, all of which are pathologically characterized by biliary tract impairment and bile retention. Intriguingly, we did not observe any decrease in Cdc42 expression or obvious aberrant expression and distribution of junction and polarity proteins in these diseased livers (Figure 6C-D, Figure S7), which suggested that the decrease of Cdc42 in the BA liver is unlikely to be caused by bile toxicity but rather by a preexisting defect.

### Whole-exome sequencing suggests a Cdc42 signaling defect in BA liver

It is unclear why Cdc42 decreases in the BA liver. Using peripheral blood cells, numerous BA patient samples have been sequenced to reveal a genetic defect. We speculated that specific mutations might be occurring in the liver that might not be detected in peripheral blood cells; therefore, we performed whole-exome sequencing of nine biopsied BA livers.

**Figure 7:**
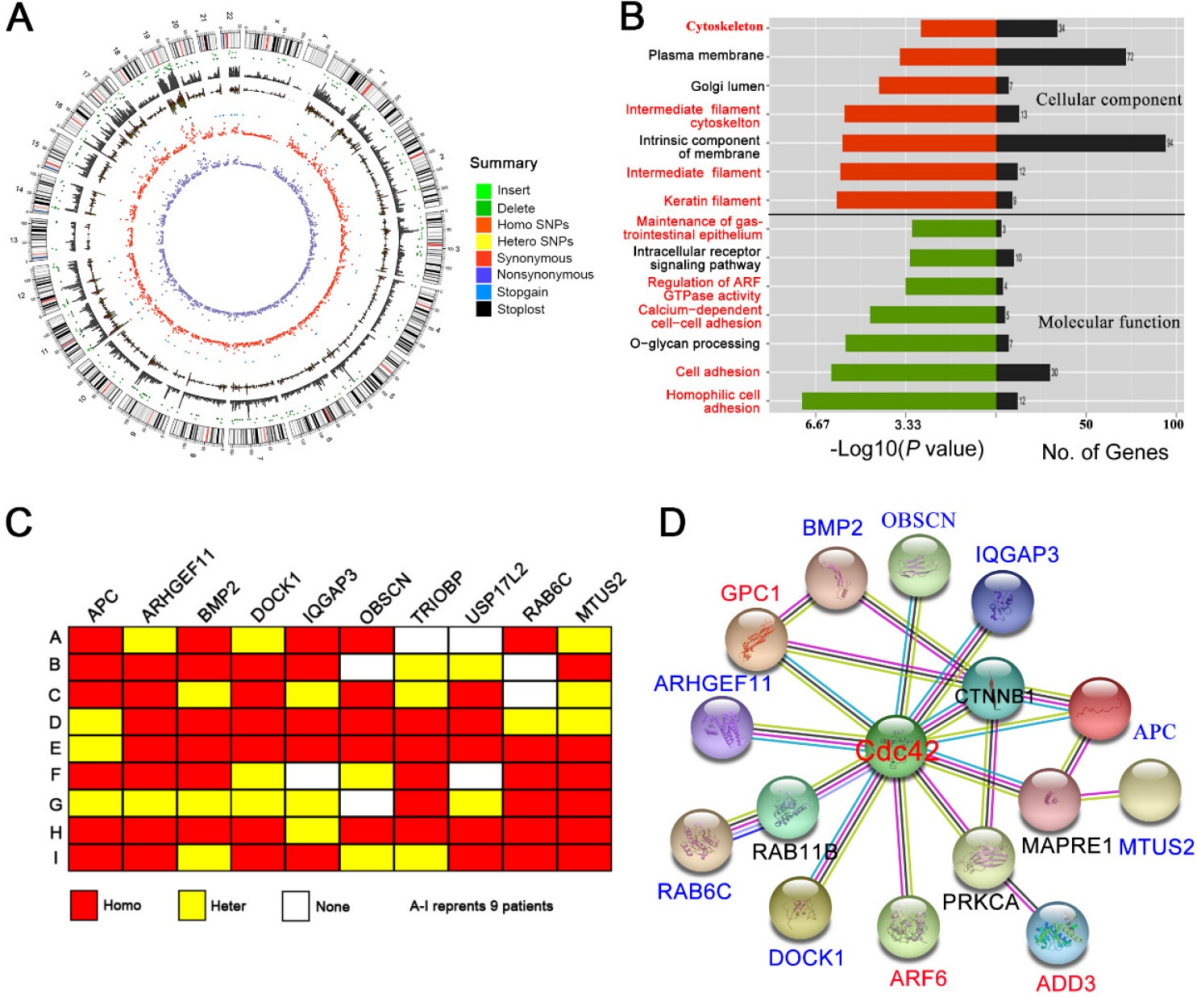
** WES suggests a Cdc42 signaling defect in the BA liver. (A)** Summary of the WES results from one BA child obtained using Circos. The different color dots indicate the different types of genetic mutations on each chromosome. The outermost circle represents the chromosomes, arranged in a clockwise direction. The position of the centromere is indicated by a red line; the gray rectangle in the second outer circle shows the coverage of chromosome reads; the dark green dot between the two circles is the missing site, and the light-green dot is the insertion site. The other types of mutations from outside to inside are: homozygous SNPs (orange rectangle), heterozygous SNPs (yellow rectangle), stop-loss mutation (black dot) and stop-gain mutation (blue dot), synonymous mutation (red), and nonsynonymous mutation (purple). **(B)** GO analysis of the cellular component and molecular function categories of the genes potentially related to BA pathogenesis in nine BA children. **(C)** Schematic map of 10 gene mutations in the livers from nine BA children. Homo, homogeneous change; Hetero, heterogeneous change; None, no change. **(D)** The interactions of genes potentially related to BA pathogenesis with Cdc42 were analyzed using the String online platform.

Approximately 90,000 single nucleotide polymorphism (SNP) loci were detected in each BA liver (Figure 7A; Table S3). The pathogenicity of the SNP loci was further analyzed by comparison with the 1000Genome, dbSNP138 and ESP6500 databases. The prediction algorithms of Mutation Taster, LRT, SIFT, Polyphen-2, PhyloP, and the GERP++ databases, were also utilized. We screened hundreds of potentially pathogenic genes that may be associated with BA. Cluster analysis showed that most of these genes were related to the cytoskeleton, cell adhesion and cell junctions (Figure 7B), which are the basis of cell polarity. Nine potential SNP loci with high frequency mutations were further identified, five of which correspond to the APC, ARHGEF11, BMP2, IQGAP3, and USP17L2 genes, which have been previously identified in BA patients. The other four SNP loci correspond to the DOCK1, OBSCN, RAB6C and MTUS2 genes, which were identified here for the first time (Figure 7C; Table S4).

Using the protein interaction analysis platform String (http://string-db.org), we found that eight of the nine identified high-frequency mutant genes have direct or indirect (but still close) interactions with Cdc42. In addition, the reported BA-related genes, GPC1, ADD3 and ARF6, were also associated with Cdc42 (PPI enrichment, *P* value = 4.47 × 10^-8^) (Figure 7D). Although we did not observe Cdc42 mutations in BA livers, the Cdc42-associated signaling network was definitely disrupted, which might have led to the decrease in Cdc42 expression.

## Discussion

The etiology of BA is far from clear, hindering its prenatal diagnosis and early treatment. In the current study, we found that Cdc42 and its active form Cdc42-GTP were robustly decreased in BA livers, accompanied by the disruption of epithelial junctions and cell polarity. Loss of Cdc42 in the bile duct epithelium led to histological changes similar to those found in BA patients. Our study also suggested the important role of bile acids in aggravating damage to genetically defective biliary cells.

So far, Babu et al. reported that cell junction defects were observed in BA patients and cultured cholangiocyte spheres 44, while the correlation between these defects and BA development remains unknown. Gene sequencing analysis has suggested that genes associated with tight junctions and adherens junctions are enriched in aberrantly expressed gene sets 45; however, to the best of our knowledge, we have reported in detail for the first time that tight junctions and polarity complexes between parenchymal liver cells, including hepatocytes and cholangiocytes, are severely disrupted in BA livers. Tight junctions and polarity complexes are structures that play fundamental roles in maintaining the barrier and transport of bile. An incomplete barrier leads to bile corrosion of the epithelium and submucosal tissues, thereby destroying the lumen structure, which further aggravates cholestasis and liver injury. Interestingly, proteins involved in tight junctions and polarity complexes, such as ZO-1, Par6, Par3 and E-cadherin, were not significantly decreased in the BA liver, but their distributions on the cell surface were extremely disordered. The correct assembly of the junction and polarity complexes is strictly orchestrated by Cdc42. Indeed, we found that the protein expression levels of Cdc42 and its active form GTP-Cdc42 were significantly decreased in all of the included cases.

We established two types of transgenic mice to elucidate the role of Cdc42 in BA development. In the albumin-Cre-based Cdc42 gene-deficient mouse whose Cdc42 gene was constitutively ablated both in hepatocytes and intrahepatic biliary cells, the hepatocytes showed the same defect in cell junctions and polarity as in cholangiocytes, which is consistent with that in human BA livers. However, this model was limited by the intact extrahepatic bile duct. We next established inducible cholangiocyte-specific Cdc42-knockout mice in which the extrahepatic bile ducts developed progressive damage and obstruction after birth. In contrast to human BA livers, the junction and polarity complexes in the hepatocytes remained intact in this model. Neither of our models completely replicated the histopathological changes in the human BA liver; nevertheless, our models better replicate the histology and progression of human BA than existing models do, such as an RRV-induced BA model that lacks obvious junction and polarity defects in the diseased liver. Most importantly, our work revealed the critical role of Cdc42 in BA development.

One of the reasons for excluding BA as a genetic disease is because there is evidence that the genetic defect occurs in all cells, while the severe injury only takes place in the biliary tree and other epithelial cells, such as the pancreatic duct, are seldom involved. Interestingly, in 70-80% of BA children, no similar pathological changes have been observed in the epithelium of other organs or tissues 46. One of the most interesting findings in this study was the profound disruption of cell junctions and polarity in both hepatocytes and cholangiocytes in human BA livers. Although it has been established that bile acids can alter hepatocyte polarity *in vitro* 47 and that TJs are impaired in a few chronic cholestatic liver diseases, we did not observe severe disruption of either cell junctions or polarity in hepatocytes and cholangiocytes, nor did we observe Cdc42 deficiency, secondary to bile retention. These findings demonstrate that the unique structural defects in BA liver cells are more likely caused by a genetic disorder.

It is interesting that little impairment was observed in the pancreas, since Cdc42 was simultaneously deleted in the pancreatic ducts in our inducible Cdc42 ablated mouse, and the pancreatic juices might be more erosive than bile acids. Decreasing the bile production could delay the progress of extrahepatic duct damage, suggesting a unique role of bile toxicity in the development of BA. Another question is why extrahepatic bile ducts are more sensitive to bile toxicity, as intrahepatic biliary cells and hepatocytes have the same genetic and structural background. We postulate that the bile acids in the extrahepatic ducts are more concentrated (i.e., bile acids are greater than ten times more concentrated in the gallbladder) and might be more toxic than those in the upstream ducts. This hypothesis requires further investigation.

The reason for the decrease in Cdc42 has not been identified. We did not find any potential pathogenic mutation in Cdc42 per se, but in nine BA liver biopsies we did detect many significant mutations in genes that are predicted to be involved in Cdc42 signaling. These genes either regulate Cdc42 transcription or participate in the assembly and maintenance of the cytoskeleton, cell junctions and polarity. Because the decline of GTP-Cdc42 was found to be parallel to that of total Cdc42, the decreased Cdc42 signaling is unlikely caused by the aberrant activation of Cdc42.

Taken together, our findings reveal that Cdc42 deficiency, even if not the only or most critical reason, plays an important role in the development of BA by impairing the assembly of cell junction and polarity complexes. We also identified that bile acids are critical for accelerating BA development. The clinical application of examining Cdc42 activity for the early diagnosis and treatment of BA patients requires further investigation.

## Supplementary Material

Supplementary figures and tables.
