# Supplementary material for: Congenital biliary atresia is correlated with disrupted cell junctions and polarity caused by Cdc42 insufficiency in the liver
Source: Theranostics. 2021 May 24;11(15):7262–75. doi: 10.7150/thno.49116 (PMC8210598; doi:10.7150/thno.49116)
Supplement: Supplementary file 1 — Supplementary figures and tables. [file thnov11p7262s1.pdf]

SUPPLEMENTARY MATERIAL

Supplementary methods

Liver function

The serum liver enzymes alanine aminotransferase (AST), aspartate aminotransferase (ALT), and serum bilirubin were assessed via standard automated techniques using a Synchron CX5 Pro analyzer (Beckman Coulter, Fullerton, CA). The concentration of cytokines in the mouse serum, including INF-γ, IL-17A, IFN-γ, and TNFα, were measured by bead-based multiplex assays using the Luminex technology according to the manufacturer's protocol (Millipore, Massachusetts, USA).

Cholangiocyte isolation

Single liver cells were isolated by two-step collagenase digestion and gradient centrifugation. Briefly, the mouse was first anesthetized by methoxyflurane and then implanted with an indwelling needle in the portal vein. Liver was perfused with buffer A (NaCl, 8.3 g/L; KCl, 0.5 g/L; HEPES, 2.4 g/L; EGTA, 0.95 g/L; Heparin, 30 u/mL PH=7.4) for 3-5 min and then subjected to buffer B perfusion (NaCl, 3.9 g/L; KCl, 0.5 g/L; HEPES, 24 g/L; CaCl2*2H20, 0.7 g/L; DNase, 0.5mg/mL; PH=7.6) for 10 min by a micro pump at a speed of 5 mL/min. Buffers were preheated at 37 °C. After perfusion, liver was removed out and put into a 10mm petri dish and incubated in 37 °C incubator for further digestion (5 min). Liver envelope was removed by micro tweezers, and liver tissue was washed with PBS solution. And then liver cells' suspension was obtained. Cholangiocytes were pre-enriched by Percoll gradient centrifugation. To capture $CK19^+$ cholangiocytes, the cells were labeled with primary CK19 antibody and subsequently magnetically isolated using the EasySep PE Selection Kit (StemCell Technologies, Vancouver, Canada) according to the manufacturer's instructions. All procedures were conducted as previously described [1].

Histology, immunohistochemistry and immunofluorescence staining

Mouse livers harvested at defined points and human liver samples were fixed with 10% buffered formalin for 48 h and then processed for sectioning by embedding in paraffin.

Paraffin sections (4 μm) were prepared for H&E, Masson, Sirius Red and immunohistochemistry staining. In addition to some special requirements, the fresh liver samples were further fixed overnight in 10% buffered formalin, washed with PBS and dehydrated in 30% sucrose at 4 °C overnight. They were then embedded in OCT. Sections (4 μm) were prepared for immunofluorescence staining. The detailed procedure was carried out as previously described. [(1)] The antibodies used in this study are listed in Supplementary Table 1.

Visualization of the biliary tree

To visualize the mouse intrahepatic biliary tree with confocal scanning, the nuclei, hepatocytes and biliary tree were labeled with three fluorescent dyes, namely, Hoechst, rhodamine B and FITC, respectively. The detailed experimental procedures were carried out as previously described [2]. A fluorescent dye mixture containing rhodamine B (1 mg/ml) and Hoechst (1.5 mg/ml) was intraperitoneally injected at a dose of 2 ml/kg body weight. Twenty minutes after the injection, the mouse was anaesthetized with ether, the abdominal cavity was opened, and the common bile duct was ligated. The whole liver was then removed without being injured or scratched. A FITC solution (1 mg/ml) was slowly infused into the intrahepatic biliary tract by retrograde injection from the gallbladder with a 26-gauge needle and a micro pump at a speed of 1 ml/h. The injection was terminated immediately before the FITC solution reached the surface and edge of the liver. Injection of excess mixture might lead to FITC leaking into the portal vein, and further injection would lead to leakage into the parenchyma. Thus, the optimal amount of this fluorescent solution for livers from normal 8-12-week-old mice is typically approximately 30-60 μl. The gallbladder was ligated immediately after removal of the needle to avoid circumfluence. The liver was then washed several times with PBS to remove any leaked or scattered dye, and the whole liver was scanned within 1 h. The materials used in this experiment are listed in Supplementary Table S2.

References

1. Ji H, Zhou Y, Zhuang X, Zhu Y, Wu Z, Lu Y, et al. HDAC3 Deficiency Promotes Liver Cancer

through a Defect in H3K9ac/H3K9me3 Transition. Cancer Res. 2019;79(14):3676-3688.

2. Chen Y, Bai L, Zhou Y, Zhang X, Zhang J, Shi Y. Fine-scale visualizing the hierarchical structure of mouse biliary tree with fluorescence microscopy method. Biosci Rep. 2020;40(5):BSR20193757

Supplementary Figures

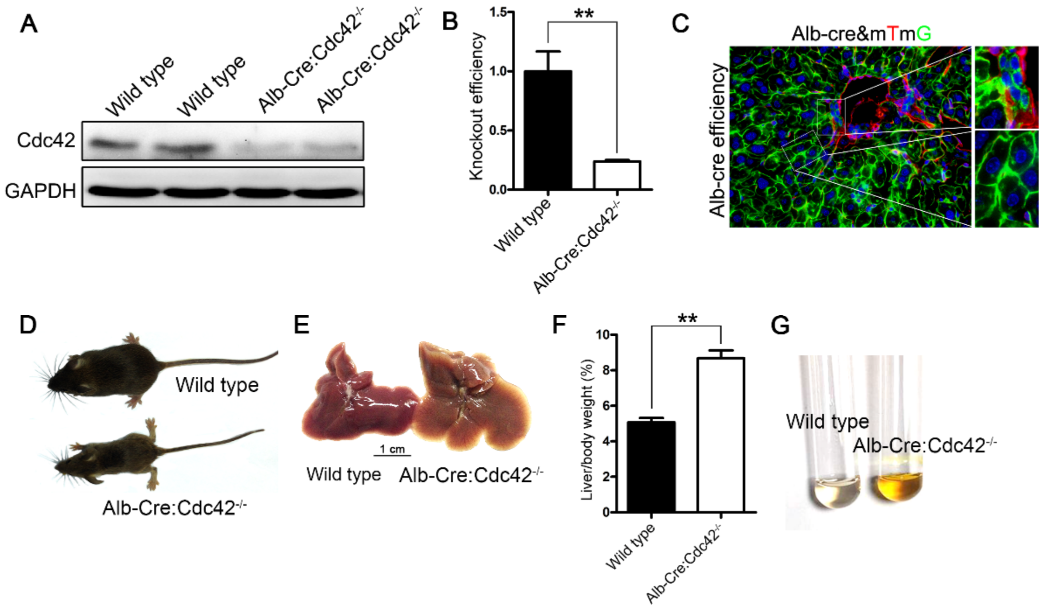

Figure S1. Construction of *Alb-Cre:Cdc42*$^{-/-}$ mice. (A) Immunoblotting was performed using total protein lysates from wild type and *Alb-Cre:Cdc42*$^{-/-}$ livers. GAPDH served as the loading control. **(B)** The knockout efficiency was analyzed according to the expression level of Cdc42 in wild type and *Alb-Cre:Cdc42*$^{-/-}$ livers. **(C)** Albumin-cre recombination efficiency was further measured by crossing with fluorescent reporter transgenic mice (mTmG); cells expressed EGFP instead of red fluorescent protein (Tomato) when the Cre-mediated recombination took place. **(D-E)** Macroscopic views of wild type and *Alb-Cre:Cdc42*$^{-/-}$ 2-month-old mice and liver showed the growth retardation and hepatomegaly caused by Cdc42 deletion. **(F-G)** Liver/body weight ratio and serum color of each genotypic mouse type. The data are presented as the mean ± s.e.m.; the *P* value was determined by a two-tailed t-test.

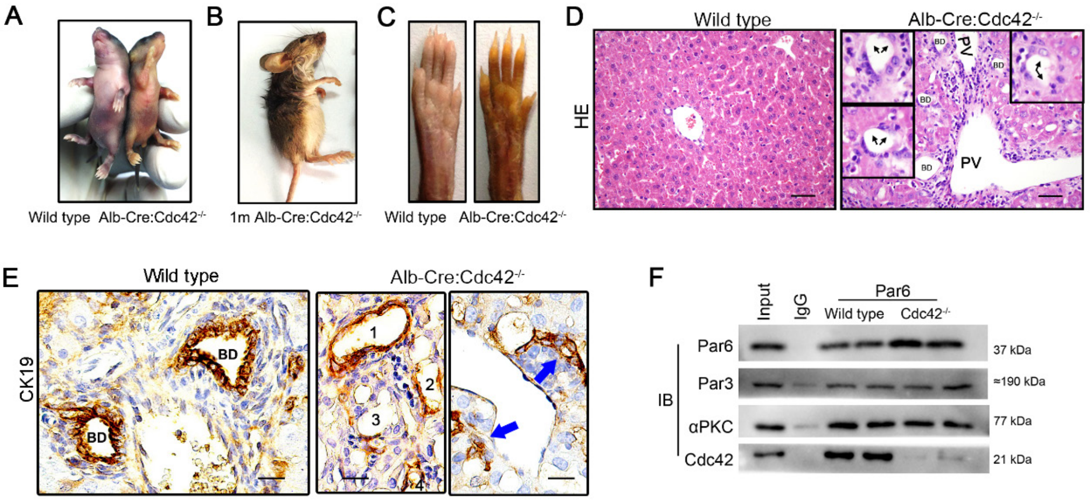

Figure S2. Liver-specific Cdc42 deletion leads to severe liver injury and bile duct destruction in mice. (A) Macroscopic views of wild type and *Alb-Cre:Cdc42*$^{-/-}$ mice at 7 days after birth. **(B)** Macroscopic view of dead *Alb-Cre:Cdc42*$^{-/-}$ mice aged 4 weeks. **(C)** Macroscopic view of 2-month-old wild type and *Alb-Cre:Cdc42*$^{-/-}$ mouse palms. **(D)** H&E-stained liver sections. The black arrowheads indicate non-continuous biliary epithelium. BD, bile duct; PV, portal vein. Scale bar, 50 μm. **(E)** CK19 staining in liver sections of wild type and *Alb-Cre:Cdc42*$^{-/-}$ mice. Arabic numerals (1-4) show altered or damaged bile ducts, and blue arrows indicate an absence of bile duct lumens. BD, bile duct; PV, portal vein. Scale bar, 50 μm. **(F)** Co-immunoprecipitation (Co-IP) assay detected in Cdc42 *Alb-Cre:Cdc42*$^{-/-}$ mouse liver.

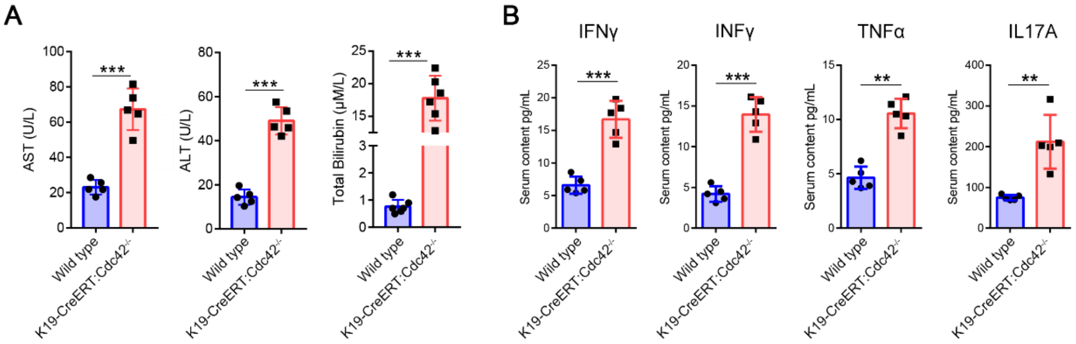

Figure S3. Serological indices of wild type, *K19-CreERT:Cdc42*$^{-/-}$ mice. **(A)** Hepatic enzymes were measured (n=5), ***, $P < 0.001$. The data are presented as the means ± s.e.m.; the P value was determined by the two-tailed Student's t test. **(B)** BA-related inflammatory factors were highly elevated in *K19-CreERT:Cdc42*$^{-/-}$ mouse serum detected by bead-based multiplex assays (n=5). **, $P < 0.01$; ***, $P < 0.00$.

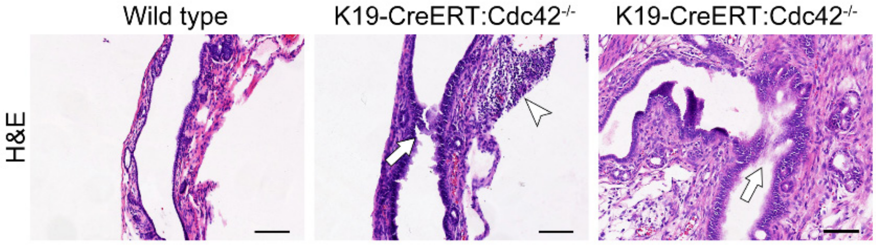

Figure S4. H&E staining of extrahepatic bile duct (EHBD) in wild type mice (left), and EHBD (middle) and hilar bile duct (right) of K19-CreERT:Cdc42-/- mice. Scale bar, 100 μm.

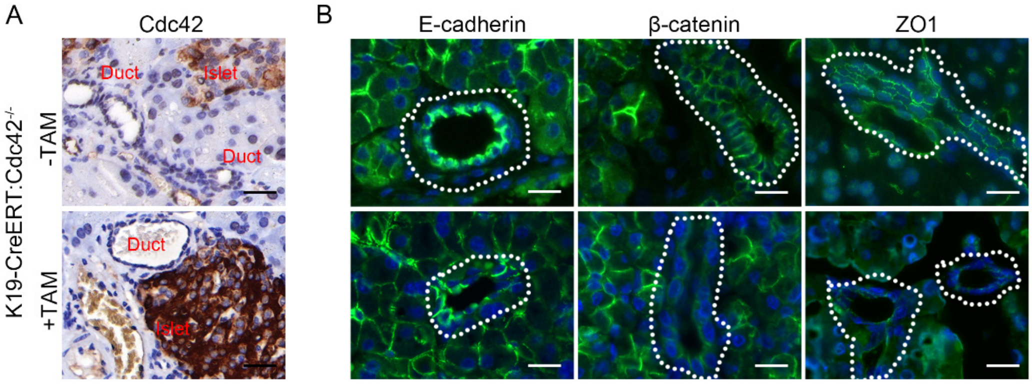

Figure S5. The influence of inducible Cdc42 ablation in pancreas. (A) Cdc42 was specifically ablated in pancreas duct epithelial cells. Scale bar, 50 μm. (B) Cdc42 ablation disrupted the distribution of E-cadherin, β-catenin and ZO1 in pancreatic ductular epithelium. Scale bar, 25 μm.

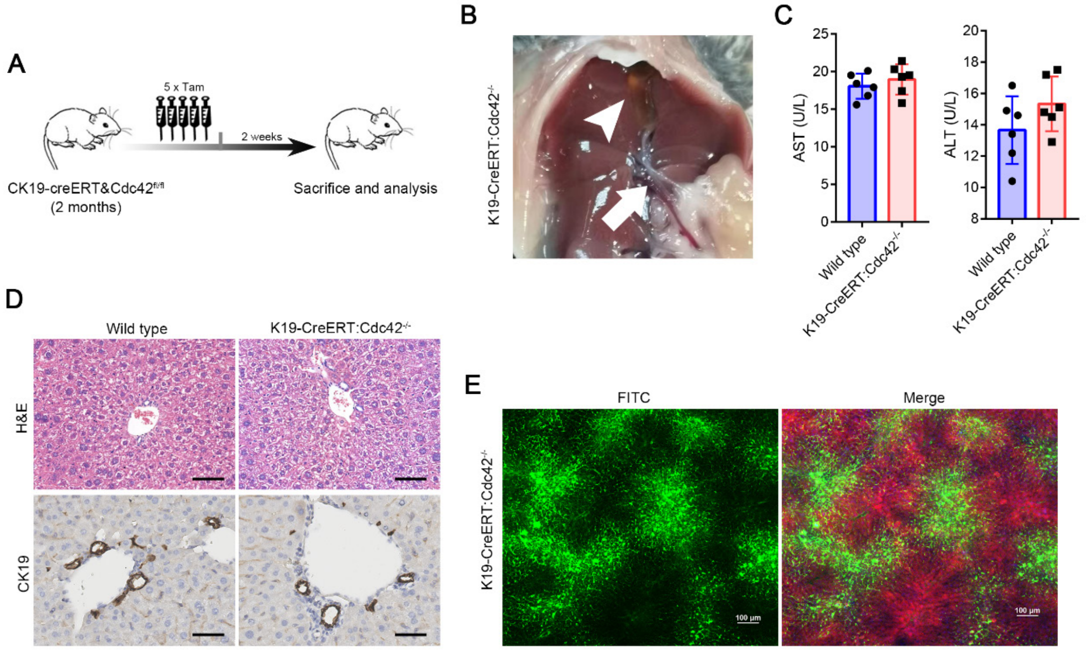

Figure S6. Inducible Cdc42 ablation has little effect on mature liver. (A) A brief diagram depicting the strategy of evaluating the effect of ablating Cdc42 in mature mouse. **(B)** Anatomical views of gallbladder (white arrow head) and extrahepatic bile ducts (white arrow) of *K19-CreERT: Cdc42*$^{-/-}$ mice. **(C)** Hepatic enzymes were measured after Cdc42 deleted in mature mouse cholangiocytes. (n=6), **, $P < 0.01$. The data are presented as the means ± s.e.m.; the *P* value was determined by the two-tailed Student's t test. **(D)** Liver histologic examination by H&E and CK19 staining after Cdc42 deleted in mature mouse cholangiocytes. Scale bar, 50 µm. **(E)** The visualization of intrahepatic bile tracts of *K19-CreERT: Cdc42*$^{-/-}$ mice after Cdc42 deleted in mature mouse cholangiocytes by retrograde cholangiography. Scale bar, 100 µm.

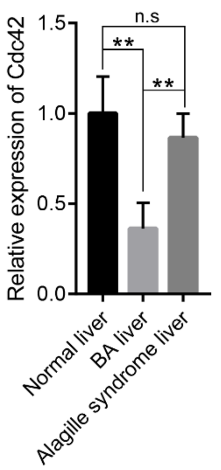

Figure R7. Cdc42 mRNA was detected by qRT-PCR. **, *P*<0.01; n.s, not significant.

Supplementary Tables

Supplementary Table 1. Antibodies

Antibody	Company	Identifier
Cdc42	Abcam	Cat# ab41429
Cdc42	Abcam	Cat# ab155940
ZO-1	Novus	Cat# NBP1-85047
ZO-1	Life	Cat# 617300
ZO-1	Invitrogen	Cat# 339188
β-Catenin	CST	Cat# 8480
β-Catenin	Abcam	Cat# ab22656
E-cadherin	CST	Cat# 3195S
P120	Life	Cat# 33-9700
Par6	Abcam	Cat# ab180159
Par3	Novus	Cat# NBP1-88861
Claudin3	Abcam	Cat# ab52231
αPKC	Abcam	Cat# ab32376
F4/80	Thermo	Cat# MA5-16632
CK19	Abcam	Cat# ab133496
CK19	Novus	Cat# NBP2-34379
Ki67	Thermo	Cat# RM-9106-S1

Supplementary Table 2. Reagents and Kits

Reagent	Company	Identifier
Hoechst 33342	Thermo	Cat# H1399
FITC	Sigma	Cat# 74817
Sulforhodamine B	Sigma	Cat# 230162
Cholic acid	Sigma	Cat# V900488
3,5-diethoxycarbonyl-1,4-dihydrocollidine	Sigma	Cat# 137030
Cdc42 Activation Assay Biochem Kit (20 Rxns)	Cytoskeleton	Cat# BK034-S
MILLIPLEX MAP Mouse TH17 Magnetic Bead Panel - Immunology Multiplex Assay	Millipore	Cat# MTH17MAG-47K

Supplementary Table S3: Summary of SNP Loci and Indels

Categories		A	B	C	D	E	F	G	H	I
SNP	Total	81820	85063	85203	92501	84866	90987	89868	78699	90557
	Known	80894	84095	84233	91435	83866	89925	88850	77821	89475
	Novel	926	968	970	1066	1000	1062	1018	878	1082
	Homo	39781	41571	41495	43405	41049	43407	44678	38320	43092
	Heter	42039	43492	43708	49096	43817	47580	45190	40379	47465
Indel	Total	7306	7450	7872	8928	7633	8699	8357	6619	7983
	Known	6702	6766	7185	8042	6979	7934	7633	6106	7227
	Novel	604	684	687	886	657	765	724	513	756

SNP, single nucleotide polymorphism; **Indel**, insertion/deletion; **Homo**, homogenous; **Heter**, heterogenous.

Supplementary Table S4: Summary of Gene Mutations in 9 BA Patients

Gene	CHR	Position	Ref	Alt	A	B	C	D	E	F	G	H	I	PhyloP	SIFT
**BMP2**	chr20	6759115	A	T	1\1	1\1	0\1	1\1	1\1	1\1	0\1	1\1	0\1	0.999086	1
**TRIOBP**	chr22	38119213	G	A	-	0\1	0\1	1\1	1\1	1\1	1\1	1\1	0\1	0.99698	0.926
	chr22	38121152	C	A	-	0\1	-	0\1	1\1	1\1	1\1	1\1	0\1	0.910881	0.936
**IQGAP3**	chr1	156518379	G	A	1\1	1\1	0\1	1\1	-	-	0\1	0\1	0\1	0.83367	0.96
	chr1	156526387	C	G	-	-	0\1	-	1\1	0\1	-	0\1	1\1	0.998803	1
**USP17L2**	chr8	11995062	G	A	-	0\1	1\1	1\1	1\1	-	0\1	1\1	1\1	0.950572	0.97
	chr8	11995540	C	T	-	0\1	1\1	1\1	1\1	-	0\1	1\1	1\1	0.937631	0.94
**ARHGEF11**	ch1	156907081	T	C	0\1	1\1	1\1	1\1	1\1	1\1	0\1	1\1	1\1	0.034992	0.98
**APC**	ch5	112176756	T	A	1\1	1\1	1\1	0\1	0\1	1\1	0\1	1\1	1\1	0.844772	0.98
**DOCK1**	chr10	129249662	G	A	0\1	1\1	1\1	1\1	1\1	0\1	0\1	1\1	1\1	NA	NA
**OBSCN**	chr1	228505204	G	A	1\1	-	1\1	1\1	1\1	0\1	-	1\1	0\1	NA	NA
**RAB6C**	chr2	130738163	G	A	1\1	-	-	0\1	1\1	1\1	1\1	1\1	1\1	NA	NA
**MTUS2**	Chr13	29898768	A	C	0\1	1\1	0\1	0\1	1\1	1\1	1\1	1\1	1\1	NA	NA

Notes: CHR, chromosome; **Ref**, reference base; **Alt**, mutation base; **A-I** represent 9 BA patients; **0** represents no mutation, **1** represents 1 mutation, **-** represents no change. When the pathogenicity score calculated by the **PhyloP** database or **SIFT** database was greater than 0.95, it was considered that the loci were conserved and highly pathogenic. The algorithm of the dbNSFP database was used for correction.
